# The influence of epidural anesthesia in pregnancies with scheduled vaginal breech delivery at term: a hospital-based retrospective analysis

**DOI:** 10.1007/s00404-023-07244-w

**Published:** 2023-11-20

**Authors:** Roman Allert, Dörthe Brüggmann, Florian J. Raimann, Nadja Zander, Frank Louwen, Lukas Jennewein

**Affiliations:** 1 Department of Obstetrics and Gynecology, University Hospital Frankfurt, Goethe University, 60590 Frankfurt, Germany; 2 Department of Anesthesiology, Intensive Care Medicine and Pain Therapy, University Hospital Frankfurt, Goethe University, 60590 Frankfurt, Germany; 3 Department of Midwifery Frankfurt, Goethe University, 60590 Frankfurt, Germany

**Keywords:** Breech, Labor, Anesthesia, Epidural

## Abstract

**Introduction:**

Epidural anesthesia is a well-established procedure in obstetrics for pain relief in labor and has been well researched as it comes to cephalic presentation. However, in vaginal intended breech delivery less research has addressed the influence of epidural anesthesia. The Greentop guideline on breech delivery states that there’s little evidence and recommends further evaluation.

**Objective:**

The aim of this study was to compare maternal and neonatal outcomes in vaginally intended breech deliveries at term with and without an epidural anesthesia.

**Design:**

This study was a retrospective cohort study.

**Sample:**

This study included 2122 women at term with a singleton breech pregnancy from 37 + 0 weeks of pregnancy on and a birth weight of at least 2500 g at the obstetric department of University hospital Frankfurt from January 2007 to December 2018.

**Methods:**

Neonatal and maternal outcome was analyzed and compared between women receiving “walking” epidural anesthesia and women without an epidural anesthesia.

**Results:**

Fetal morbidity, measured with a modified PREMODA score, showed no significant difference between deliveries with (2.96%) or without (1.79%; *p* = 0.168) an epidural anesthesia. Cesarean delivery rates were significantly higher in deliveries with an epidural (35 vs. 26.2%, *p* = 0.0003), but after exclusion of multiparous women, cesarean delivery rates were not significantly different (40.2% cesarean deliveries with an epidural vs. 41.5%, *p* = 0.717). As compared to no epidurals, epidural anesthesia in vaginal delivery was associated with a significantly higher rate of manual assistance (33.8 versus 52.1%) and a longer duration of birth (223.7 ± 194 versus 516.2 ± 310 min) (both *p* < 0.0001)".

**Conclusion:**

Epidural anesthesia can be offered as a safe option for pain relief without increasing neonatal or maternal morbidity and mortality. Nevertheless, it is associated with a longer birth duration and manually assisted delivery.

## What does this study add to the clinical work

**Table:** 

Epidural anesthesia can be offered as a safe option for pain relief for women attending vaginal breech birth without increasing neonatal or maternal morbidity or mortality. Also, cesarean delivery is not associated with an epidural anesthesia. Women should be informed that epidural anesthesia is associated with manually assisted delivery and a prolonged first and second stage of labor.	

## Introduction

Regional anesthesia is a well-established procedure in obstetrics for pain relief in labor and is broadly recommended in guidelines [1]. A Cochrane review including data of 40 trials and over 11.000 women shows a higher chance of instrumental assisted delivery in trials before 2005, an effect that did not occur when trials before 2005 were excluded from the analysis. No difference was shown concerning neonatal outcome or the rate of cesarean delivery [2].

In deliveries with breech presentation evidence is scarce regarding the safety and effect of epidural anesthesia and recommendations are vague: the British Greentop guideline states that the effect of an epidural anesthesia on the success of vaginal breech birth is unclear and might increase the risk of intervention and recommends further research [3]. The French clinical practice guideline emphasizes the high level of evidence for epidural anesthesia in cephalic version, with no higher risk of cesarean or risk of vaginally assisted delivery and therefore encourages the use of epidural anesthesia in breech presentation [4]. The SOGC (Society of Obstetricians and Gynecologists of Canada) clinical practice guideline on breech delivery recommends avoiding dense epidural to maximize expulsive efforts, while neither ACOG (American College of Obstetricians and Gynecologists) nor RANZCOG (Royal Australian and New Zealand College of Obstetricians and Gynaecologists) addresses the issue of epidural anesthesia [5–7].

In the term Breech trial epidural anesthesia was not associated with adverse perinatal outcome [8–10]. The PREMODA (PREsentation et MODe d'Accouchement) trial does not report an impact of epidural anesthesia [11]. Even though safety of epidural anesthesia is established, there still are reports of associated increased adverse neonatal outcome, prolonged labor, or cesarean delivery rate [12–14].

In the FRABAT (FRAnkfurt Breech At Term Study Group) cohort, the demand for epidural analgesia was high, especially in primiparous women [15]. Thus, it can be assumed that the patients’ need for an epidural anesthesia during an intended vaginal breech birth is high and clinicians will be confronted with this topic frequently during clinical counseling.

Since every medical intervention with its possible complications should be discussed with patients before administration, it is mandatory to gain evidence in order to be able to give reliable information. The effect of epidural analgesia on vaginally intended birth out of breech presentation has not been elucidated properly because the respective recommendations are adopted from vertex presentations. We present a cohort study on the neonatal and maternal outcome in vaginally intended breech deliveries in light of the use of an epidural anesthesia. We hypothesize that an epidural anesthesia does not influence perinatal morbidity in vaginally intended breech deliveries provided the epidural keeps the motor function and patients are not immobilized.

## Methods

### Study design

We conducted a single center cohort study in all pregnant women at term (≥ 37 weeks of gestation) presenting with a breech presentation at the Goethe University Hospital Frankfurt, Germany, from January 2004 to December 2018. The analysis was performed in a retrospective manner through generating subgroups (deliveries with or without an epidural) within our study cohort.

The university hospital’s ethics committee gave consent (420/11). All data were assessed through the in-house patient data system as well as the Hessen Perinatalerhebung and were acquired after patient’s dismissal from the hospital. All patients received the standard clinical care. Because of the retrospective nature of data acquisition, the ethics committee waived an informed patient’s consent.

Exclusion criteria were fetal birth defect, uterine malformation, multiple pregnancies, contraindication for an epidural anesthesia, estimated birth weight less than 2500 g, and contraindications for vaginal approach.

Other studies with intersection cohorts have been published by different authors of the FRABAT group within previous publications. [15–19].

### Clinical procedure and counseling

All pregnant women with a breech presentation are counseled between 34 and 36 weeks of gestation. External cephalic version, vaginal attempted birth, as well as cesarean delivery are discussed with each patient, depending on the individual patient history and examination. During vaginal delivery, which is performed predominantly in an upright maternal position, manual assistance to deliver the arms or the fetal head is performed by a trained physician if necessary. A maternal upright position applies when the mother stands or is on all fours (hands and knees). An epidural is offered to every woman by their own choice if no contraindications (e.g., thrombocytopenia) are present. Counseling specifics and details on manual assistance in the upright maternal position have been published [17, 20]

### Outcome parameters

Primary outcome was perinatal fetal morbidity, which was assessed using the modified PREMODA Score, potentially associated with the delivery mode. The PREMODA Score is adapted from the PREMODA study [11] implies NICU stay > 4 days, trauma at birth, neurological deficits, intubation > 24 h, or an APGAR score of less than 4 at 5 min [9]. Secondary outcome measures were duration of labor, rate of cesarean delivery, and rate of assisted vaginal delivery.

### Method of epidural anesthesia

Epidural anesthesia was administered by an in-house anesthesiologist. It was initiated with a dose of Ropivacaine and Sufentanil. After the loading dose, a patient controlled pump with Ropivacaine / Sufentanil was connected to maintain persistent pain reduction. Patients were not immobilized and the rate could be reduced if necessary. If analgesia was not sufficient patients could receive up to three additional boli per hour.

### Statistical analysis

Groups of variables were tested for normal distribution with Kolmogorov–Smirnov test [21]. Group differences were analyzed using Pearson’s χ2 testing. Student’s T-test was utilized to compare continuous variables [22, 23]. A nominal logistic regression analysis with Wald testing was performed.[24] We used JMP 14.0 software (SAS Institute, Cary, NC, USA) for our analyses. A p-value of below 0.05 was considered as statistically significant.

## Results

Of the 2122 women presenting for counseling with breech presentation at our center, 1413 attempted vaginal delivery.

744/1413 (52.7%) women received an epidural anesthesia (EPI group), 669/1413 (47.3%) did not (NEPI group, Table 1). Patients in the NEPI group were significantly older than patients in the EPI group (NEPI 32.7 (± 4.5), EPI 31.9 (± 4.3) *p* = 0.0009). BMI was equally distributed between both groups (Table 1). There were significantly more primiparous women in the EPI group (EPI 523, 70.3%; NEPI 316, 47.2%; *p* < 0.0001; Table 1). Mean birth weight was significantly higher in the EPI group (3388 g; NEPI: 3323 g; *p* = 0.002; Table 1). Duration of pregnancy was significantly longer in the EPI group (280 days) as compared to the NEPI group (278 days, *p* > 0.0001; Table 1).

**Table 1:** Vaginally intended deliveries (*n* = 1413) with (n = 744) and without (*n* = 669) epidural anesthesia

Characteristic	No epidural (*n* = 669) NEPI	Epidural (n = 744) EPI	*p*-value
Age (mean, SD)	32.7 (± 4.5)	31.9 (± 4.3)	0.0009
BMI (mean, SD)	23.2 (± 4.1)	22.9 (± 3.7)	0.9508
Parity (n, %)			< 0.0001
1	316 (47.23%)	523 (70.30%)	
> 1	353 (52.77%)	221 (29.70%)	
Birth weight (grams, mean ± st. dev.)	3323 (± 16)	3388 (± 15)	0.002
Systemic disease (high blood pressure, hypothyroidism and others)	106 (15.84%)	112 (15.05%)	0.7125
Duration of Pregnancy (days, mean ± std. dev.)	277.5 (± 8.7)	280.4 (± 7.9)	< 0.0001
Cesarean delivery	175 (26.2%)	260 (35.0%)	0.0003
Vaginal birth	327 (48.9%)	232 (31.2%)	< 0.0001
Manually assisted	167 (25.0%)	252 (33.9%)	< 0.0001
Arterial pH < 7.0	2 (0.3%)	6 (0.8%)	0.2931
5 min APGAR < 4	3 (0.45%)	4 (0.54%)	1,0000
NICU > 4 days	25 (3.74%)	44 (5.91%)	0.0639
Intubation > 24 h	5 (0.75%)	8 (1.08%)	0.5864
Perinatal infection	17 (2.54)	38 (5.11%)	0.0132
PREMODA	27 (4.04%)	47 (6.32%)	0.0565
PREMODA possibly related to delivery mode	12 (1.79%)	22 (2.96%)	0,1677

**Fig. 1:**
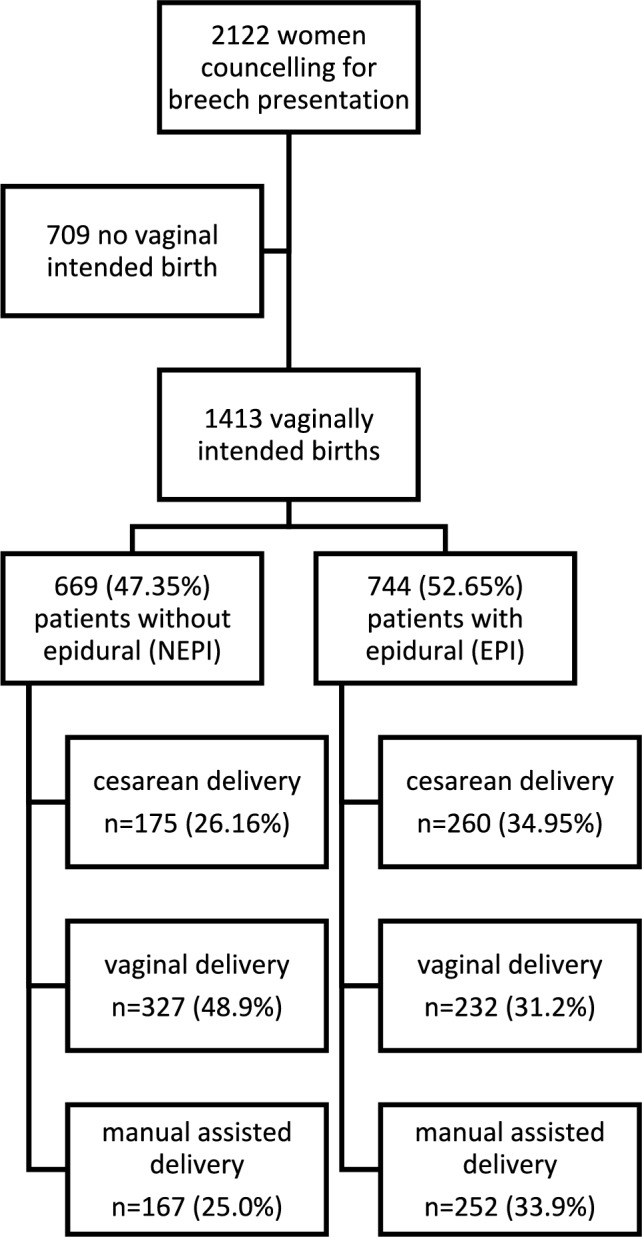
Flow chart of the study cohort

There were significantly more manually assisted vaginal deliveries when women received an epidural anesthesia: In the NEPI group 327/669 (48.9%) women delivered vaginally, while 167/669 (25.0%) delivered with manual assistance. In the EPI group 232/744 (31.2%) women delivered spontaneous and 252/744 (33.9%) with assistance (*p* < 0.0001). Cesarean delivery after onset of labor was performed in 175/669 (26.2%) in the NEPI group which is significantly less often than in the EPI group (260/744 (35.0%), *p* = 0.0003, Table 1 and Fig. 1).

We investigated all vaginal deliveries in a sub-cohort analysis. There were significantly more primiparous women in the group of patients giving vaginal birth with an epidural anesthesia (vEPI group, *n* = 313, 64.7%) as compared to primiparous women without an epidural anesthesia (vNEPI group, *n* = 185, 37.5%; *p* < 0.0001, Table 2). Birth weight was not significantly different between vNEPI group (3307 ± 340 g) and vEPI group (3325 ± 391 g; *p* = 0.361; Table 2). Duration of labor was significantly longer in vaginal deliveries with an epidural anesthesia as compared to vaginal deliveries without epidural anesthesia (vEPI 516 ± 310 min; vNEPI 224 ± 194 min; *p* < 0.0001, Table 2). Manual assistance was significantly more often necessary in vaginal deliveries with an epidural anesthesia (vEPI: *n* = 252, 52.1%; vNEPI: *n* = 167 33.8%; *p* < 0.0001, Table 3). Fetal morbidity measured with the modified PREMODA score was not significantly different between both groups (vNEPI: 2.02%, vEPI: 3.31%; *p* = 0.2373; Table 2). There was no significant difference in high grade perineal tears between groups (vNEPI: *n* = 8; 1.6%, vEPI: *n* = 10; 3.3%, *p* = 0.642; Table 2), but perineal tears of all degrees were significantly more often in vaginal deliveries with an epidural anesthesia (vNEPI: *n* = 224; 45.3%, vEPI: *n* = 249; 51.4%, *p* = 0.0056; Table 2).

**Table 2:** Vaginal deliveries (*n* = 978) with (*n* = 484) and without (*n* = 494) an epidural anesthesia

Characteristic	No epidural (*n* = 494) vNEPI	Epidural (*n* = 484) vEPI	*p*-value
Parity (*n*, %)			< 0.0001
1	185 (37.45%)	313 (64.67%)	
> 1	309 (62.55%)	171 (35.33%)	
Birth weight (grams, mean ± st. dev.)	3306.8 (± 340)	3325.3 (± 391)	0.3606
Duration of first stage of labor (minutes ± std. dev.)	193.9 (± 184)	434.3 (± 291)	< 0.0001
Duration of second stage of labor (minutes ± std. dev.)	28.3 (± 33.9)	80.0 (± 81,9)	< 0.0001
Duration of labor (minutes ± std. dev.)	223.7 (± 194)	516.2 (± 310)	< 0.0001
Spontaneous birth	327 (66.2%)	232 (47.9%)	< 0.0001
Manually assisted	167 (33.8%)	252 (52.1%)	< 0.0001
PREMODA	16 (3.24%)	28 (5.79%)	0.0640
PREMODA possibly related to delivery mode	10 (2.02%)	16 (3.31%)	0.2373
Perineal tear (all degrees)	224 (45.34%)	249 (51.45%)	0.0056
High grade perineal tear (III°, IV°)	8 (1.62%)	10 (2.07%)	0.6415
Episiotomy	11 (2.23%)	17 (3.51%)	0.2539

**Table 3:** Comparison of primiparous women with a fetus in breech presentation and the intention to deliver vaginally with (*n* = 523) or without (*n* = 315) epidural

Characteristic	No epidural (*n* = 316) pNEPI	Epidural (*n* = 523) pEPI	*p*-value
Birth weight (grams, mean ± st. dev.)	3252.6 (± 411)	3378.9 (± 416)	< 0.0001
Cesarean delivery	131 (41.46%)	210 (40.15%)	0.7174
PREMODA	13 (4.11%)	40 (7.65%)	0.0414
PREMODA possibly related to delivery mode	5 (1.58%)	20 (3.82%)	0.0917

We investigated a subgroup of primiparous women (*n* = 839). In the group of primiparous women with an epidural anesthesia (pEPI) birth weight was significantly higher as compared to deliveries of primiparous women without an epidural anesthesia (pNEPI: 3253 ± 411 g, pEPI: 3379 ± 416 g; *p* < 0.0001, Table 3). Cesarean delivery rate was not significantly different between groups in this sub-analysis (pNEPI: *n* = 131; 41.5%, pEPI: *n* = 210; 40.2%; *p* = 0.7174, Table 3). In primiparous women, there was no significant difference in the modified PREMODA score whether patients received an epidural or not (pNEPI: *n* = 5; 1.58%, pEPI: *n* = 20 3.82%; *p* = 0.0917, Table 3).

Within a multiple nominal logistic regression analysis, maternal age, birth weight, neonatal morbidity, and cesarean delivery were not significantly associated with an epidural anesthesia (Table 4). In contrast, primiparity (OR 2.295; 95% CI: 1.781–2.956; *p* < 0.0001) and pregnancy duration (OR 1.316; 95% CI: 1.182–1.465; *p* < 0.0001) were significantly associated with an epidural anesthesia (Table 4).

**Table 4:** Nominal logistic regression model of risk factors associated with epidural anesthesia in women with breech presentation and the intention to deliver vaginally (*n* = 1413)

Dependent variable: Epidural anesthesia	Odds ratio	95% Confidence interval	*P*-value
Maternal age (years)	0.975 per Unit	0.950–1.002	0.074
Birth weight (kilograms)	1.072 per Unit	0.778–1.477	0.671
Pregnancy Duration (weeks)	1.316 per Unit	1.182–1.465	< 0.0001
Primiparity	2.295	1.781–2.956	< 0.0001
Mod. PREMODA possibly related to delivery mode	1.523	0.679–3.414	0.307
Cesarean Delivery	1.177	0.905–1.532	0.225
Sub-analysis within the group with final vaginal delivery without or with manual maneuvers (*n* = 978)			
Duration of birth (minutes)	1.0055 per Unit	1.0044–1.0066	< 0.0001
Manually assisted delivery	2.34	1.57–3.52	< 0.0001
Perineal injuries	1.38	0.93–2.06	0.112

In the subgroup of vaginal deliveries, only duration of birth (OR 1.0055; 95% CI: 1.0044–1.0066; *p* < 0.0001) and manually assisted delivery (OR 2.23; 95% CI: 1.57–3.52; *p* < 0.0001) were significantly increased, whereas perineal injuries were not affected (Table 4).

## Discussion

Evidence is scarce on the impact of an epidural anesthesia in vaginally intended breech deliveries since all recommendations are based on studies investigating epidural analgesia in cephalic deliveries. We have performed a cohort study on vaginally intended breech deliveries analyzing the effect of epidural anesthesia on perinatal outcome.

Perinatal morbidity was not significantly different between deliveries with and without epidural anesthesia (see Tables 1, 2, 3, 4). Furthermore, Goffinet et al. [11] showed that increased short-term morbidity in breech deliveries did not translate into long-term morbidity. Primiparous women were analyzed separately because parity has an impact on delivery outcome measures. In our sub-cohort analyses of primiparous women (Table 3) and a nominal logistic regression model (Table 4), we were able to confirm the data seen in our whole cohort analyses concerning fetal morbidity. Here, PREMODA scores were consistently not different between deliveries with and without epidural anesthesia.

Patients receiving an epidural anesthesia had a higher probability for cesarean delivery after onset of labor in our main cohort (Table 1). But when only primiparous women were analyzed, cesarean delivery rates were not significantly different (Table 3). Also, a nominal logistic regression analysis found no association of cesarean delivery rate and epidural anesthesia (Table 4). The effect on cesarean delivery rates thus derives from the influence of parity. Primiparous women received an epidural anesthesia in 70.3% of cases, multiparous women only in 29.7% (Table 1). This finding contrasts the RCOG guideline; here authors stated that an epidural *“might increase the risk of caesarean section”*[3]. In vertex deliveries, a Cochrane analysis reports no effect on cesarean delivery rates linked to the use of epidurals [2].

New data suggest that not the epidural anesthesia but a prolonged labor and higher need for pain relief itself pose risk factors for an increased cesarean delivery likelihood; underlying problems are the actual cause rather than the analgesia itself [25].

In vaginal deliveries, the duration of the labor was significantly longer in deliveries with an epidural anesthesia. This effect has also been reported in vertex deliveries [26, 27]. In these studies the immobilization though the application of an epidural is supposedly causative for a longer birth duration. In our center, patients are not immobilized after they receive pain relief by an epidural. This is important because women give birth predominantly in an upright position in order to reduce interventions and newborn morbidity [20]. This is both arguable in vertex and breech presentations. We believe that a “walking” epidural—keeping maternal motor function—is of important benefit for the course of labor: walking and an upright position reduce the duration of labor and the risk of cesarean [28].

Among the patients who delivered vaginally epidural anesthesia was associated with a higher chance of assisted vaginal delivery (see Tables 1, 4). From vertex deliveries we have learned that operative vaginal deliveries are more often performed in deliveries with an epidural anesthesia [26].

When a vaginal operative delivery is indicated because of arrest of birth in active labor, women without an epidural anesthesia might prefer a cesarean section, while women with an epidural anesthesia might feel more equipped for a vaginal operative procedure.

In the cohort of women who experienced a successful vaginal breech delivery, maternal morbidity was not significantly increased in patients with an epidural anesthesia; in particular, we did not find a higher rate of third- and fourth-degree perineal tears or tear of all degrees (Tables 2 and 4). Our data imply that the use of an epidural for patients with a breech presentation undergoing labor is safe and not associated with a higher morbidity – neither for the fetus nor for the mother.

A strength of our study is a large cohort of patients, treated with a standardized protocol. This leads to homogeneity and comparability within our results.

A major limitation of our study is selection bias as all data derive from a single center. This is a retrospective analysis of an existing study cohort. Thus, only associations and not causative relationships can be concluded from our data. A prospective randomized controlled trial would be the gold standard to investigate a clinical intervention. Nevertheless, randomized controlled trials are hardly possible in women with breech delivery and an intention to deliver vaginally since only a few women would accept to stay without pain relief and to withhold an epidural due to a study design would be unethical.

In our data, only the application of an epidural analgesia was documented. The degree of actual pain relief and the time point of administration during labor were not recorded. Duration of pain relief of an epidural analgesia and patient satisfaction are important issues possibly influencing our outcome measures. In future studies, these items should be assessed in order to improve the quality of our results.

However, while the retrospective analysis has limitations, the absence of an influence on perinatal morbidity in our study adds value to the body of knowledge: our data show that mothers will not impact perinatal morbidity by requesting an epidural during labor, contrasting studies by Macharey or Toijonen. In these studies an epidural has been associated with adverse perinatal outcome in breech deliveries [12, 14].

As in vertex presentations, an epidural anesthesia may be offered to ensure pain relief and is a safe gold standard for analgesia during labor. If manual assistance during birth is necessary, a sufficient pain relief might also be beneficial.

Further research in prospective settings would provide a more robust foundation for clinical decision-making and improve the understanding of the impact of epidural anesthesia on breech deliveries.

## Data Availability

The data that support the findings of this study are available from the corresponding author, [RA], upon reasonable request.
